# Cell-of-Origin and Genetic, Epigenetic, and Microenvironmental Factors Contribute to the Intra-Tumoral Heterogeneity of Pediatric Intracranial Ependymoma

**DOI:** 10.3390/cancers13236100

**Published:** 2021-12-03

**Authors:** Tiziana Servidei, Donatella Lucchetti, Pierluigi Navarra, Alessandro Sgambato, Riccardo Riccardi, Antonio Ruggiero

**Affiliations:** 1 Pediatric Oncology Unit, Fondazione Policlinico Universitario Agostino Gemelli IRCCS, Catholic University of the Sacred Hearth, 00168 Rome, Italy; riccardi@me.com (R.R.); Antonio.Ruggiero@unicatt.it (A.R.); 2 Dipartimento Universitario di Medicina e Chirurgia Traslazionale, Università Cattolica del Sacro Cuore, 00168 Rome, Italy; donatella.lucchetti@unicatt.it (D.L.); alessandro.sgambato@unicatt.it (A.S.); 3 Department of Healthcare Surveillance and Bioethics, Section of Pharmacology, Università Cattolica del Sacro Cuore-Fondazione Policlinico Universitario A. Gemelli IRCCS, 00168 Rome, Italy; Pierluigi.Navarra@unicatt.it; 4 Centro di Riferimento Oncologico della Basilicata (IRCCS-CROB), 85028 Rionero in Vulture, PZ, Italy

**Keywords:** intra-tumoral heterogeneity, ependymoma, genetics, epigenetics, tumor microenvironment, cancer stem cells, single cell RNA seq, H3K27me3, H3K27M

## Abstract

**Simple Summary:**

Intra-cranial ependymoma (EPN) accounts for approximately 10% of pediatric brain tumors. The current therapeutic strategies have not significantly improved prognosis, which is still dismal in nearly 40% of patients. Major challenges for treatment are chemorefractoriness of EPN, tendency to recur, and high intra-tumoral heterogeneity (ITH). It is increasingly emerging that stalled neurodevelopmental programs driven by cancer stem cells (CSCs)/progenitor cells are at the root of oncogenesis and ITH of pediatric brain tumors, including EPN. This is the first review that examines how genetic and heritable epigenetic alterations and environmental selection forces drive ITH of pediatric intra-cranial EPN in the perspective of the CSC model. This review also summarizes how improvement in the single-cell technology has deepened the comprehension of the complexity, cell-of-origin, and developmental trajectories of EPN, paving the way for novel therapeutic options.

**Abstract:**

Intra-tumoral heterogeneity (ITH) is a complex multifaceted phenomenon that posits major challenges for the clinical management of cancer patients. Genetic, epigenetic, and microenvironmental factors are concurrent drivers of diversity among the distinct populations of cancer cells. ITH may also be installed by cancer stem cells (CSCs), that foster unidirectional hierarchy of cellular phenotypes or, alternatively, shift dynamically between distinct cellular states. Ependymoma (EPN), a molecularly heterogeneous group of tumors, shows a specific spatiotemporal distribution that suggests a link between ependymomagenesis and alterations of the biological processes involved in embryonic brain development. In children, EPN most often arises intra-cranially and is associated with an adverse outcome. Emerging evidence shows that EPN displays large intra-patient heterogeneity. In this review, after touching on EPN inter-tumoral heterogeneity, we focus on the sources of ITH in pediatric intra-cranial EPN in the framework of the CSC paradigm. We also examine how single-cell technology has shed new light on the complexity and developmental origins of EPN and the potential impact that this understanding may have on the therapeutic strategies against this deadly pediatric malignancy.

## 1. Introduction

Tumors are complex ecosystems composed of non-malignant and malignant cell populations [1]. The malignant populations themselves are genetically and phenotypically heterogeneous and define the so-called intra-tumoral heterogeneity (ITH) that governs tumor evolution [2] and drug resistance [3]. Although ITH is a “contemporary concept” [4], its complex nature was highlighted back in the 1970s [5]. However, the mechanistic explanations and full understanding of the origins of ITH still have a long way to go.

The first model to explain ITH was the clonal evolution model, whereby stochastic DNA mutations confer a growth advantage to single cancer cells, which are selected for and clonally outcompete the other cells [6,7], overcoming spatial and temporal microenvironmental constraints in a Darwinian-like process [8]. Cancer cells may also adapt epigenetically to ever-changing tumor niches by virtue of high intrinsic cellular plasticity [9,10].

Besides the gene-centric view, another framework of ITH is the cancer stem cell (CSC) model, whereby a small subpopulation of cells become hierarchically organized, phenotypically diverse tumor cells or, alternatively, shift reversibly between stem-like and more committed cell states (CSC plasticity model) [11,12]. It is likely that the clonal evolution model and the CSC model coexist and act together in a cooperative manner to determine ITH.

In this light, tumors might be considered as an “organ system”, where the cellular subclones act as “tissue types” with distinct functions [13] and reciprocal signaling between tumor subpopulations [14,15,16] and between tumor and the surrounding microenvironment [17], with complex interactions to enhance tumor fitness and facilitate immune evasion [18], drug resistance [19], and metastasis [20].

**Figure 1:**
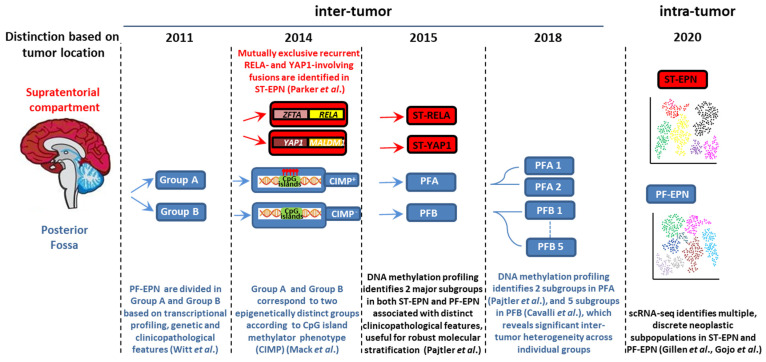
A timeline of the most important molecular findings which have contributed to uncovering inter-tumor and intra-tumor heterogeneity in intracranial pediatric ependymoma.

Pediatric brain tumors (PBTs) represent the leading cause of cancer-related morbidity and mortality in children [21]. The second most common PBT is ependymoma (EPN), a group of molecularly and clinically heterogeneous entities that in children arise almost exclusively intra-cranially. Despite advances in the understanding of EPN biology, the prognosis is still grim in approximately 40% of patients because of a high degree of ITH and intrinsic chemoresistance [22]. Over the last few years, whole-genome sequencing, gene-expression profiling, and genome-wide methylation at a whole-population level have stratified EPN into nine molecular groups, four of which represent the major types of intracranial pediatric EPN and differ in demographic, clinicopathological, and (epi)genetic profiles (Figure 1) [23].

ST-EPN-RELA (ST-RELA) and ST-EPN-YAP1 (ST-YAP1) tumors arise in the supratentorial (ST) compartment, and are distinguished by mutually exclusive recurrent *zinc finger translocation associated (ZFTA)-RELA proto-oncogene, NF-kB subunit (RELA)* or *Yes1 associated transcriptional regulator (YAP1)-involving fusions* [24], whereas PF-EPN-A (PFA) and PF-EPN-B (PFB) tumors occur in the posterior fossa (PF) [25].

To date, the majority of research and treatment decisions in EPN have been based on analyses of bulk tumors that, however, return an averaged picture of all cell populations. The advances of sequencing technologies and single-cell omics over the last decade have deepened the knowledge of the bewildering heterogeneity of the tumor genome, transcriptome, epigenome, and proteome at an unprecedented scale [26,27].

After briefly summarizing EPN inter-tumoral heterogeneity, the goal of this review is to synthesize how the main genetic, epigenetic, and environmental factors drive ITH in pediatric intracranial EPN in the light of the CSC model. We also examine how single-cell RNA sequencing (scRNA-seq) has contributed to the understanding of the complexity and developmental origins of EPN, unraveling some common transcriptional programs across different EPN subtypes, and even across different pediatric brain tumors, which might help define potential druggable vulnerabilities.

## 2. Inter-Tumoral Heterogeneity and Clinicopathological Characteristics of Pediatric Intracranial EPN: A Brief Overview

EPNs are neuroepithelial tumors that account for approximately 10% of pediatric intra-cranial neoplasms. Surgery and radiotherapy are well-established treatments, whereas chemotherapy has not clearly demonstrated a survival benefit. According to the World Health Organization (WHO) classification for tumors of the central nervous system (CNS), the four major pediatric EPN entities are WHO grade II/III [28], although high ITH makes accurate grading challenging. The predominant groups in children are highly aggressive PFA and ST-RELA EPNs, whereas PFB and ST-YAP1 show an indolent behavior with a more favorable outcome [23]. Although molecular subgrouping shows better correlation with prognosis than histological grade alone, to date it has not informed treatment strategies [22], which have remained substantially unchanged over recent years and unsuccessful in approximately 40% of patients [21]. Very recently, these four EPN molecular variants have been incorporated into the fifth edition of the WHO Classification of Tumors of the CNS published in 2021 (WHO CNS5) [29], which integrates molecular diagnostics with more traditional histopathological approaches. According to WHO CNS5As, ST EPNs are now categorized into two types containing either *ZFTA* (formerly, *C11orf95*) fusions (because *ZFTA* may be fused with partners others than *RELA*), or *YAP1* fusions.

Approximately 70% of childhood EPNs occur in the PF, whereas 20% occur in the supratentorium. PFAs arise in younger children (<5 years) and are characterized by dysregulation of numerous cancer-related networks, such as angiogenesis, receptor tyrosine kinase (RTK) signaling, and cell cycle (Table 1) [25]. Despite an aggressive behavior, PFAs display a balanced genetic profile and absence of highly recurrent oncogenic events. The most common copy number alterations are 1q gain [23] and 6q loss [23,30], both associated with unfavorable outcome. Instead, epigenetic modifications are hallmarks of PFAs, including CpG island methylator phenotype (CIMP) [31], DNA hypomethylation [32] and global reduction of the repressive histone H3 lysine 27 trimethylation (H3K27me3), with focal retention of H3K27me3 at CpG islands [32,33,34]. Recently, other alterations of the epigenetic landscape of PFA have been discovered, including infrequent histone H3K27M mutation (with a lysine 27-to-methionine exchange) [35], *enhancer of zeste homologs inhibitory protein (EZHIP*, formerly *CXorf67*) mutations, and overexpression of *EZHIP* in nearly all the cases [36] (see Section 4.2.1).

PFBs frequently occur in older children (5–18 years) and display many gains and losses of entire chromosomes [23], but dysregulation of a very restricted number of pathways controlling microtubule assembly and oxidative metabolism [25] (Table 1). DNA methylation profiling has evidenced significant inter-tumor heterogeneity in both PFAs and PFBs, distinguishing further subgroups with distinct patterns of genetic alterations and expression profiling, which suggests distinct histogenesis involving a cell of origin at different anatomic locations in the hindbrain [36,37].

More than two thirds of ST-EPNs harbor alternative *ZFTA–RELA* fusions [24] (Table 1), that lead to constitutively active NF-κB signaling, an established driver of solid tumors [38]. In addition, ST-RELA EPNs display other subgroup-specific genomic alterations, including frequent loss of chromosome 9 and homozygous *INK4a-ARF (CDKN2a)* deletions [23]. *YAP1* fusions, the most common being *YAP1-mastermind like domain containing 1 (MAMLD1)*, define the other clinically relevant subgroup of ST-EPN, rare tumors with relatively stable genomes, besides recurrent rearrangements involving *YAP1* gene locus on chromosome 11 [23,39]. Compared to ST-RELA, *YAP1-MAMLD1* tumors differ in demographic distribution (occurring mainly in children with a median age of 1.4 years vs. 8 years of RELA EPNs, and mostly restricted to female patients), anatomical location (intra-/periventricular in YAP1 vs. cerebral in RELA) and prognosis (favorable vs. unfavorable) [40].

There is increasing evidence of ST-EPNs with alternative gene fusions and ambiguous DNA methylation-based classification [41,42,43]. Pediatric supratentorial *RELA* fusion-negative EPNs show other fusion events, the majority involving *ZFTA* as a partner gene, such as *ZFTA-mastermind like transcriptional coactivator 2 (MAML2)* or *ZFTA-nuclear receptor coactivator 1 (NCOA1)*. These tumors exhibit histopathological heterogeneity, no nuclear NF-κB expression, and epigenetic proximity to the *RELA* methylation class in some cases [44,45]. Recently, recurrent fusions involving the *pleomorphic adenoma gene-like 1* (*PLAGL1*) gene have been discovered in a group of histopathologically diagnosed ST EPNs exhibiting a distinct DNA methylation profile [46], that occur mostly in children. To add complexity, rearrangements of *ZFTA* associated with *RELA* DNA methylation profiling have also been identified in EPN with an infratentorial location [47,48].

**Table 1:** Summary of the main genetic/epigenetic alterations and clinicopathological characteristics of the major groups of pediatric intra-cranial EPN.

Molecular Group	ST-RELA	ST-YAP1	PFA	PFB	References
Location	ST, cerebral	ST, intra-periventricular	PF	PF	[25,40]
Age	children/adolescents median age 8 years	young children median age 1.4 years	young children median age 3 years	all age groups median age 30 years	[23]
Gender					[23]
Male	65%	25%	65%	41%	
Female	35%	75%	35%	59%	
Molecular events					
Genetic	chromothripsis *ZFTA-RELA* fusions *CDKN2a* deletion loss of chromosome 9	*YAP1*-fusions	balanced genome 1q gain 6q loss infrequent H3K27M substitution infrequent *EZHIP* mutations	chromosomal instability	[23] [23,24] [23,30] [35] [36]
Epigenetic			CIMP positive DNA hypomethylation H3K27me3 loss EZHIP overexpression	CIMP negative H3K27me3 retention	[31] [32] [32,34] [36]
Pathogenic impact	NF-κB pathway cell cycle cell migration MAPK pathway	Hippo pathway	angiogenesis RTK pathways cell cycle cell migration derepression of PRC2 target genes	ciliogenesis oxidative metabolism	[24,39] [23,25] [31]
Outcome	poor	favorable	poor	favorable	[23]

## 3. CSCs as a Source of ITH

### 3.1. The CSC Model

The CSC model was revived about two decades ago with the isolation of a subset of functionally distinct cells from hematologic and solid malignancies that uniquely drive tumor growth [49], and has rapidly emerged to explain the versatile features of tumor populations [50]. The isolation of clonogenic neural stem cells (NSCs) from human fetal brain tissue [51] corroborated the hypothesis that brain tumors may develop from transformed NSCs or progenitor cells [52].

CSCs are rare, relatively quiescent cells endowed with indefinite self-renewal, multilineage differentiation properties, and tumorigenicity, whereas transiently proliferating, more differentiated, non-tumorigenic non-CSCs form the bulk tumor [49,53]. Brain tumor SCs (BTSCs) are also functionally identified based on their ability to propagate serially in an undifferentiated state and to form floating clonally derived heterogeneous colonies called neurospheres (NSs) [54]. Central to the CSC paradigm is the identification of cancer-specific markers that allow unequivocally distinguishing of CSCs from non-CSCs. However, some controversies over universal stemness markers exist. For example, the cell surface glycoprotein CD133 has been proposed as a robust marker for BTSCs, although CD133-negative populations exhibit stemness functional features [55], and other proteins, such as nestin and SOX2, define the BTSC immunophenotypic profile [9], consistent with remarkable context-dependent inter-cellular heterogeneity among the BTSC population itself.

**Figure 2:**
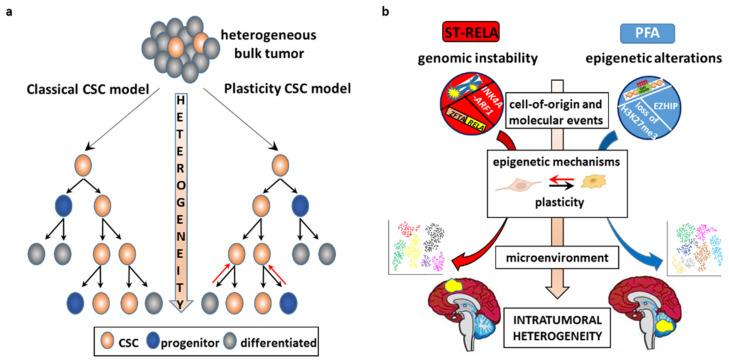
Intra-tumoral heterogeneity (ITH) of EPN in the cancer stem cell (CSC) perspective. (**a**) Schematics of the classical (on the left) and plasticity (on the right) CSC model. The classical CSC model asserts that CSCs are a rare fraction of the bulk tumor, able to self-renew and to differentiate along multiple lineages, driving unidirectional hierarchy of heterogeneous cells. CSC plasticity model proposes that cancer cells can dynamically transition from a CSC state to a non-CSC state and vice versa. Combinatorial dynamics of genetic and epigenetic alterations and distinct microenvironmental contexts engender cell stemness and plasticity, installing ITH. (**b**) CSC model(s) in intra-cranial ependymomagenesis. Molecular events occurring in distinct cells of origin drive ependymomagenesis in the two compartments: genomic instability, leading to chromothripsis, gene fusions, and *INK4a-ARF* deletions, predominate in ST-RELA, whereas epigenetic factors (e.g., CIMP phenotype, global loss of H3K27me3, *EZHIP* overexpression) predominate in PFA.

According to the classical paradigm, CSCs divide asymmetrically and give rise to daughter stem cells and non-stem progeny to drive unidirectional hierarchy-organized phenotypic differentiation that installs ITH (Figure 2a). More recently, accumulating evidence has revisited the traditional model into the plasticity model [12], whereby cancer cells possess the ability to bidirectionally transition between an SC and non-SC state. According to this model: (1) CSCs are not necessarily a small, slow-proliferating fraction of the bulk tumor, and (2) the functional features of both CSCs and non-CSCs are dynamically generated by combinatorial genetic and non-genetic factors. The observation that purified breast cancer [56] and glioblastoma (GBM) [9,57] stem and non-stem cells re-establish an equilibrium of mixed populations of all cellular states in cultures and in vivo is a proof of principle of the plasticity model.

Although cancers result from genetic and epigenetic events in interaction with the microenvironment, the cell(s) where these events occur are equally important determinants of tumorigenesis [58,59]. It is likely that CSCs derive from neoplastic transformation of healthy SCs, because pathways involved in the normal SC homeostasis are often hijacked and/or epigenetically altered in CSCs to bring about a “malignant reprogramming” that locks the cells into a state of self-renewal that persists beyond the timeframe of normal development (Figure 2b) [60]. Aberrant DNA hypermethylation in promoter regions displays a clonal pattern, being present in CSCs and their offspring [61], which suggests that epigenetic alterations play a causative role from very early tumorigenesis steps on and are then maintained. Alternatively, oncogenic lesions may be acquired by committed non-CSCs that undergo dedifferentiation to generate CSCs and initiate tumorigenesis [50,62].

Several lines of evidence have demonstrated that normal stem and progenitor cells are particularly susceptible to oncogenic transformation. Concurrent Ras overexpression and Tp53 inhibition transform NSCs and oligodendrocyte precursor cells (OPCs) that develop GBM-like tumors in the mouse brain, but fail to transform differentiated astrocytes [63]. Transgene expression of H3K27M is oncogenic in vivo when expressed in NSCs, but not always in OPCs [64,65,66]. By contrast, medulloblastomas with similar molecular features can be initiated by activation of Hedgehog signaling via genetic deletion of its receptor coding gene *Ptch1* in NSCs or neuronal progenitors [67]. Hence, the transcriptional context in the cell(s) of origin governs susceptibility to transformation and acquisition of tumor-initiating capability in some cancers, while in some others, driver mutations, rather than the cell of origin, appear to dictate the tumor features [58] (see Section 3.2 and Section 5.2 for EPN cell of origin).

### 3.2. CSC-Driven Preclinical Models of EPN

The relevance of CSCs in ependymomagenesis was first highlighted by the isolation from EPN of rare populations of cells with features of radial glia cells (RGCs) [68,69,70], the progenitors that give rise to neurons, astrocytes, oligodendrocytes, and ependymal cells [71]. EPN SCs express the RGC markers CD133, nestin, RC2, and brain lipid binding protein (BLBP) and fulfill all benchmark functional assays for the characterization of CSCs. In differentiation media, EPN SCs show dramatic morphological and immunophenotypic changes towards glial, neuronal, and oligodendroglial lineages as well as reduction in tumor-propagating potential [70,72], thus recapitulating the ITH of the bulk tumor and positing RGCs at the root of EPN (see Section 5.2).

Cell lines have been established from EPN surgical samples by selection of the CSC component in NS-promoting conditions [72,73,74,75]. NS models mimic a 3D structure, which resembles the tumor microenvironment (TME) more faithfully than 2D cultures, preserving cell variability [76]. Compared to cell lines grown as monolayers, EPN 3D cultures transplanted in the mouse brain show better fidelity to the original tumor in terms of genetic, transcriptomic, and histopathological characteristics [74,75].

Drug treatment of patient-derived EPN cell lines results in preferential depletion of a stem-like cell population with a tumor-initiating property, as shown by a decrease in NSC markers, increase in differentiation-associated markers, and reduction in tumorigenicity in ex vivo transplantation assays [72,74]. Specific targeting of BLBP by PPAR antagonists lessens cell migration and invasion and promotes chemoresistance in vitro [77]. In comparison with EPN stem-like cells, differentiated cells are less sensitive to temozolomide because of differentiation-induced upregulation of *MGMT* [78], although others have also reported temozolomide resistance in undifferentiated EPN SCs [74].

High cellular variability within individual EPN cell lines has been reported. For instance, serial transplantation of EPN patient-derived lines in mice [74] or cultures in medium devoid of mitogens [79] select more tumorigenic cells. Moreover, mitogen-independent EPN cell lines display constitutive activation of EGFR, AKT, and STAT3 and sensitization to EGFR inhibitors in vitro and in vivo.

Clonal expansion of patient-derived cells by differential selective pressure exerted by culture conditions can help uncover genetic ITH in EPN. One mitogen-independent highly tumorigenic EPN line has been found to harbor protein-coding *SEC61G–EGFR* fusion genes, also found in one PFA out of 16 pediatric EPN cases by RT-PCR sequencing [79] and in glioblastoma (GBM) [80]. Similar to findings in EPN, ITH of GBM with a heterogeneous pattern of expression/amplification of RTKs is revealed by genotype selection under receptor-targeted ligand stimulation [81]. GBM SCs (GSCs) in EGF-free media retain *EGFR* amplification and EGFRvIII expression, which are usually lost in cells cultured in mitogen-enriched media [82]. Together, these data suggest that differential selection in vitro and in vivo may represent a complementary strategy to address ITH and its functional relevance in EPN.

## 4. Determinants of ITH

### 4.1. Genetic ITH

As “cancer is, in essence, a genetic disease” [83], the first recognized source of ITH is the inherent genomic instability of cancer cells that generates the progressive emergence of distinct genotypes upon which selection can act in a given microenvironmental context [84]. Based on functional activity, gene mutations are mainly distinguished as driver mutations and passenger mutations [85]. Driver mutations confer a selective advantage on cancer cells by the activation of oncogenic pathways and/or the inactivation of tumor suppressors, whereas passenger mutations are neutral. However, even usually silent mutations may become advantageous in the adaptive responses to certain selection pressures, such as resource deprivation, ligand stimulation, natural defenses, or chemo/radiotherapy [7,86].

Besides small-scale genetic changes, genomic instability includes large-scale genomic events [87], such as chromosomal instability [88], aneuploidy, chromothripsis [89], and extrachromosomal DNA (ecDNA) [90], that involve an ample number of genes and provide massive copy-number amplification. Large-scale DNA alterations are quite unstable through mitoses and can undergo strong selective pressures, accelerating tumor evolution [19].

ecDNAs exist in almost 50% of tumors [91], and are circular-shaped elements of DNA with high chromatin accessibility [92] that contain amplified oncogenes and drug resistance genes [93]. As they lack centromeres, ecDNAs segregate randomly among daughter cells and confer massive intercellular genetic heterogeneity and improved fitness, that might result in tumor aggressiveness and chemoresistance [94,95]. Loss of ecDNA carrying EGFRvIII induces resistance to EGFRvIII inhibitors in GBM models and patients [96]. Different evolution of ecDNA at diagnosis and relapse has been reported across multiple cancers, including pediatric high-grade gliomas (pHGGs) [97]. However, the contribution of ecDNA to ITH of EPN has not been addressed yet.

Chromothripsis is a single cellular catastrophic event in which hundreds of genomic rearrangements take place at once in one or a few chromosomes [89]. Generally considered as an early mutational phenomenon occurring in a minority of neoplasms, sequencing-based analyses at high coverage depth have demonstrated that chromothripsis is pervasive in cancers, reaching a frequency of more than 50% in some entities [98]. Moreover, longitudinal analysis in paired primary and relapsed tumors has shown that chromothripsis may occur only in the primary tumor, only at relapse, or, conversely, in both events in the same patient, which suggests subclonal heterogeneity and evolution occurring through all steps of tumor progression [99]. A causative role for chromothripsis has been inferred in ST-RELA EPNs, where *ZFTA–RELA* fusions result from a shattering event on chromosome 11, that juxtaposes *ZFTA* to the NF-κB master transcription factor *RELA* [24] (see Section 4.1.1). Remarkably, among the nine EPN molecular subgroups, chromothripsis is detected exclusively in ST-RELA, where it more frequently involves chromosome 11 [23].

#### 4.1.1. CSCs and Genomic Instability: ST-Ependymomagenesis

Compelling evidence for a causal role of genetic alterations in CSC-driven EPN oncogenesis comes from studies in embryonic NSCs transduced with relevant EPN driver mutations, that demonstrate that regional distinct NSCs uniquely susceptible to specific mutations foster ependymomagenesis in the different anatomical compartments [100,101]. A cross-species study of human EPN and mouse NSCs isolated from different regions of embryonic and postnatal CNS with a wild-type or *Ink4a/Arf*-null genetic background has shown that the transcriptomes of human ST-EPNs with amplified *EPHB2* and deleted *INK4a*/*ARF* match only that of embryonic cerebral *Ink4a/Arf*-null NSCs [100], and neither the hindbrain nor spine. Corroboratively, *EPHB2* drives ependymal-like tumors only if expressed in embryonic cerebral *Ink4a/Arf*-null NSCs, but in neither adult NSCs nor embryonic NSCs from other CNS regions, or in the absence of *Ink4a/Arf*^−/−^ deletions.

*ZFTA–RELA* fusions have been identified as the first pathogenic genes in ST-EPN, because they are sufficient to induce EPN-like tumors in allograft models of forebrain-derived murine *Ink4a/Arf*-null NSCs, whereas neither wild-type translocation partner alone does [24]. Corroboratively, de novo ependymomagenesis has been reported in neonatal mouse brain after *RELA* fusion transfer to *NES*-expressing cells by the RCAS/tv-a system [102] or lentivirus injection [103]. Tumors develop in all models with morphological, immunophenotypic, and transcriptomic features that echo those of human ST-RELA EPNs.

Unlike wild-type RELA, RELA fusion oncoproteins are constitutively localized in the nucleus, where they drive aberrant activation of NF-κB target gene transcription in vitro and in vivo [24]. Recent key studies integrating epigenomic and transcriptomic mapping have demonstrated that a number of non-canonical NF-κB transcriptional programs, such as Notch, MAPK, and focal adhesion networks, are critical actors of ST-RELA formation, in addition to the canonical NF-κB signaling [104,105,106]. Contrary to the initial hypothesis positing that the RELA partner drives the transcriptional activity of RELA fusion proteins, recent chromatin interaction-based analyses support the idea that it is the ZFTA moiety that shuttles the RELA component to the nucleus and dictates the RELA fusion binding affinity across the genome, so as to orchestrate the transcription of ependymoma-associated genes in collaboration with RELA targets [105,106,107,108].

*YAP1* fusions involve the first exons of the *YAP1* gene fused in frame with the 3′ coding portion of other translocation partner genes, most frequently the *mastermind like domain containing 1 gene* (*MAMLD1*), and seldom the *family with sequence similarity 118 member B gene (FAM118B)* [23,24]. YAP1 functions as a transcriptional cofactor of the Hippo signaling pathway, a tumor suppressor pathway that controls organ size and tumorigenesis by sequestering YAP1 in the cytosol [109], whereas YAP1-MAMLD1 accumulates predominantly in the nucleus in ST-YAP1 tumors [39]. The oncogenicity of *YAP1-MAMLD1* and *YAP1-FAM118B* has been demonstrated via gene transfer of the full-length fusions or wild-type fusion partners to target RGCs/NSCs in the embryonal cerebral ventricular zone (VZ) using in utero electroporation [39], as well as the RCAS/tv-a system [110] or lentivirus injections [103] in mouse neonatal brains. The exogenous expression of the fusions alone drives the formation of murine tumors similar to human EPNs, identifying cells positive for the RGC marker PAX6 as the cell of origin of *YAP1-MAMLD1* EPN [39]. Interestingly, ST-YAP1 tumors display the highest PAX6 expression of all EPN subgroups [111]. In the postnatal mouse brain, constitutive expression of *YAP1-MAMLD1* impairs neural differentiation and migration of ventricular neural precursor cells that are forced instead into active proliferation. The MAMLD1 domain is necessary for translocation of the fusion in the nucleus and for the interaction with NFI transcription factors (TFs), that in turn recruit the fusion protein to enhancer regions enriched in TEAD and NFI-binding motifs to drive the transforming gene expression of YAP1-MAMLD1 EPN [39,110].

Overall, these studies demonstrate that mutually exclusive transforming fusions are likely the key event in ependymomagenesis of the ST compartment via a similar mechanism, whereby the fusion products accumulate constitutively in the nucleus of topographically restricted NSCs to disrupt developmental gene expression programs, ultimately leading to oncogenesis.

### 4.2. Epigenetic ITH

Variable phenotypes of cancer cells can also be mediated by epigenetic, transcriptional, and microenvironmental changes without concomitant genetic mutations. Non-genetic ITH is far more dynamic than genetic heterogeneity and is therefore increasingly recognized as a driving force of tumor evolution [112,113].

The term “epigenetic” describes the covalent modifications of DNA and histones that affect gene expression without intrinsic changes in the DNA sequence through modulation of the chromatin structure [112]. Epigenetic changes are inherited by offspring cells just like genetic alterations and provide an additional pool of selectable traits. An interplay between genetic and epigenetic alterations occurs in virtually all tumor types, where epigenetic lesions may precede or arise simultaneously with genetic mutations, or conversely be a consequential event [10,114]. PBTs display an overall low mutational burden, but there are a number of epigenetic dysregulations [115,116,117] that can drive tumorigenesis even in the absence of highly recurrent driver mutations, CIMP-positive PFA being a prominent example. Most of the few recurrent mutations of PBTs target epigenetic regulatory genes, such as *H3.3A, ATRX*, and *enhancer of zeste homolog 2* *(EZH2)* [118]. For example, a hallmark of pHGG is H3K27M [119], that is associated with neuroanatomical specificity, DNA methylation pattern, and age distribution [120].

Epigenome regulation of intercellular heterogeneous gene expression is a dynamic condition between transcriptionally active and repressive chromatin states by virtue of cell-to-cell variation in DNA methylation at enhancers and promoters, covalent histone modifications [33], nucleosome positioning [121], and chromatin accessibility [122]. Prominent alterations of DNA methylation in cancers, including high-risk PFAs, are focal gains at normally unmethylated CpG islands and promoter regions, that heritably silence hundreds of genes that counteract tumor development, outnumbering gene mutations [123,124]. Posttranslational covalent histone modifications include methylation or acetylation at histone tails, such as H3K27me3 and H3K27ac, markers of repressed and active transcription, respectively [114]. H3K27 trimethylation is mediated by the Polycomb repressive complex 2 (PRC2) via the methyltransferase activity of the PRC2 catalytic subunit EZH2 [125].

Epigenetic ITH has primarily been assessed focusing on DNA methylation, because of its stability and mitotic heritability, and is found in regulatory regions that control the transcription of associated genes, contributing to gene expression heterogeneity relevant to cell identity and disease processes [123,126,127,128]. High epigenetic heterogeneity at enhancers has been reported in ESCs [129] and during progression from normal tissues to primary tumors and to metastases with a cancer-specific pattern [130], which indicates that enhancer DNA methylation may be primed to respond to microenvironmental cues and to increase cancer cell plasticity. In temporally distinct tumor specimens, DNA methylation levels are reported to be increased, equal, or decreased in primary vs. relapsed tumors [131], maybe because of variable epigenetic clonal dynamics in different cancers. Compared to primary EPNs, relapsed EPNs display neither significant differences in DNA methylation profiles nor in H3K27me3 levels, whereas major changes occur at CpG islands that show higher methylations in relapsed ST-RELA and PFA EPNs [132].

Spatiotemporal epigenetic heterogeneity in distinct areas of the same tumor has been described in a wide range of cancers and allows for building the evolutionary history of the tumor alongside genetic heterogeneity. Comparison between phylogenetic and epigenetic trees has usually shown similar and integrated patterns, which suggests a codependency of genetic and epigenetic mechanisms in tumor progression [131,133]. In primary low-grade gliomas and matched recurrent HGG, cell cycle genes are epigenetically upregulated through promoter hypomethylation during tumor progression, in parallel with genetic mutations that affect cell cycle checkpoints [134]. Multiplatform molecular profiling of spatially distinct meningioma shows regional alterations in chromosome structure that underpin clonal transcriptomic, epigenomic, and histopathologic signatures [135]. DNA methylation and RNA sequencing of six topographically distinct samples from one ST-RELA tumor reveal significant transcriptional and epigenetic heterogeneity [136]. Remarkably, the expression of the subgroup-specific markers *L1CAM*, *CCND1*, *ZFTA*, and *RELA* is similar across the sections, whereas DNA methylation-based and gene expression variability define three geographically distinct clusters enriched in stem-like, neuronal differentiation, and mature microglia signatures that recapitulate brain development.

#### 4.2.1. CSCs and Epigenetic Alterations: PFA Ependymomagenesis

A link between aberrant epigenome and pediatric hindbrain tumorigenesis has increasingly been recognized [32,137]. Although the underlying mechanisms are different, they all converge on PRC2 function and targets during narrow developmental windows [138]. ESCs rely on PRC2 to reversibly silence genes required for differentiation and there is evidence that PRC2 targets similar sets of CpG-containing genes in ESCs and in cancer cells [139]. The PRC2 component EZH2 is essential for GSC maintenance and its pharmacological or molecular inhibition impairs GSC-driven tumor growth [140]. In pHGG, H3K27M competitively binds to and dominantly suppresses EZH2 function, that results in reduced H3K27me3 and spurious activation of earlier developmental programs in NSCs that are crucial for oncogenesis [141]. Some of these genes, such as *Pbx3*, *Eya1*, and *Plag1*, are regulated by bivalent promoters with both permissive and repressive histone marks that are poised for activation in stem and progenitor cell types [142]. Transgene expression of H3K27M in human ESC-derived neural progenitor cells [64] or human induced pluripotent SCs [66] synergizes with *TP53* knockout and constitutive *PDGFR* activation to enhance self-renewal and drives in vivo and de novo gliomagenesis. Concordantly, removal of H3K27M in pHGG-derived cell lines using CRISPR-Cas9 restores full differentiation capabilities along the glial lineage [143].

In PFA EPN, CpG hypermethylation is enriched at the promoter regions of genes important for neurodevelopment that are silenced by PRC2-mediated trimethylation of H3K27 in ESCs [144], such as differentiation-associated genes, thereby locking cells in a perpetual proliferative state. Corroboratively, treatment of PFA CIMP-positive cultures with demethylating agents results in derepression of the PRC2 target genes in ESCs, and impaired proliferation of PFA cells in vitro and in vivo [31].

Like H3K27M mutant pHGG, a hallmark of the PFA epigenome is global reduction in H3K27me3. However, the mechanism(s) underlying H3K27 hypomethylation in PFAs that harbor infrequent H3K27M mutations have been elusive. Recently, several publications have demonstrated that EZHIP contains a short “K27M-like” sequence that inhibits EZH2, causing reduction in H3K27me3 and an overly permissive chromatin state [145,146,147]. Transgenic cell lines with expression of either H3K27M or EZHIP exhibit comparable genome-wide loss of H3K27me3 with focal gains at CpG islands and gene expression profiles that reflect dysregulation of PRC2-mediated gene repression. Consistently, ablation of EZHIP in cell lines by a CRISPR-Cas9 strategy results in increased H3K27me3 levels [36]. However, in vivo models of EZHIP-driven EPN tumorigenesis are still lacking, suggesting that other hit(s) are required [145].

**Figure 3:**
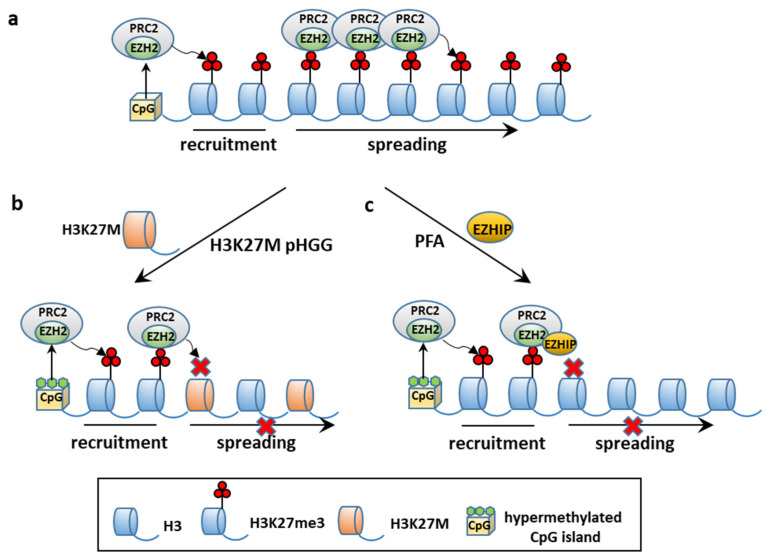
Schematics depicting mechanisms which mediate global loss of H3K27me3 in H3K27M pediatric high-grade glioma (pHGG) and PFA. (**a**) In normal cells, PRC2 is recruited to CpG islands and catalyzes H3K27 trimethylation and spreading of H3K27me3; (**b**) in H3K27M mutant pHGG, PRC2 is recruited at hypermethylated CpG islands, but H3K27M prevents spreading of H3K27Me3; (**c**) in PFA with EZHIP overexpression, PRC2 is recruited to hypermethylated CpG islands, but EZHIP competitively binds to and inhibits EZH2-mediated trimethylation of H3K27 and spreading. Both mechanisms in (**b**,**c**) determine loss of PRC2-mediated gene repression and transcription of normally silenced genes (Reprinted with permission from Siddhant U. Jain, (2020), Elsevier and Copyright Clearance Center, Licence Number 5200711451432, 2 December 2021).

Consistently with the similarities between EZHIP- and H3K27M-mediated mechanism, EZHIP overexpression shows a non-overlapping pattern with H3K27M in PFAs [36,148]. Remarkably, EZHIP missense mutations found in a small proportion of PFAs do not impair EZHIP-mediated inhibition of PRC2 activity [146]. No loss-of-function mutations of PRC2 have been found in PFA EPN and H3K27M mutant pHGG, which suggests that residual PRC2 activity is required for the development of these tumors. Cell-based and molecular assays in HGG models have shown that H3K27M and EZHIP impair the production and spread of H3K27me3 from PRC2 high-affinity sites, while sparing residual H3K27me3 at CpG sites (Figure 3) [146,149,150]. Corroboratively, removal of H3K27M restores H3K27me3 propagation associated with inhibition of cell proliferation and tumorigenicity. Analog mechanisms may occur in PFA, as hinted by the sensitiveness of PFA lines to EZH2 inhibitors [31,151].

The chromatin profile and gene signature of PFAs converge on genes involved in neurodevelopmental pathways and RGC functions [32,146]. Interestingly, during human PF neurogenesis, H3K27Me3 is reduced in RGCs in prenatal phases, while increasing postnatally [32], which indicates that dynamic gains and losses of H3K27me3 are necessary for normal neural differentiation and development. Reduced H3K27me3 in PFA tumors and in PF RGCs in early neurogenesis is consistent with RGCs as PFA presumptive cells of origin.

### 4.3. TME, CSCs, and EPN

The epigenome stands at the intersection of the genome and TME. Unlike genetic alterations, epigenetic modifications are reversible and less consistently transmitted through mitosis, and therefore play a major role in opportunistic adaptation to spatiotemporal fluctuations of the TME [3,152]. Whereas in healthy tissues the environment acts as the main barrier to counteract cancer initiation, in tumor tissues neoplastic cells subvert this organized architecture into a deranged tumor-sustaining milieu. These changes include matrix remodeling, development of tumor vasculature networks, recruitment of stromal and immune cells, and interactions between tumor and normal cells as well as between functionally different tumor subpopulations [17]. The complex tumor architecture creates topographical constraints, changeable blood flow [153], and heterogeneous microenvironmental conditions with a combinatorial dynamic of contextual cues that trigger a variety of signaling pathways and regulatory networks [13].

This paradigmatically occurs at the tumor core and tumor/host interface. Although region-specific driver mutations have been documented [154], contextual factors are equally important in shaping the zonal pattern, with high proliferation, signaling activities and invasion-promoting properties almost exclusively restricted to the leading edge of the tumor as opposed to a quiescent, apoptotic, and therapy-resistant phenotype predominating in the center. These distinct intrinsic signatures and phenotypes are driven by hypoxic [155] and/or acidic microenvironmental gradients [156,157] and paracrine cross-talk [158] between the distinct tumor populations.

**Figure 4:**
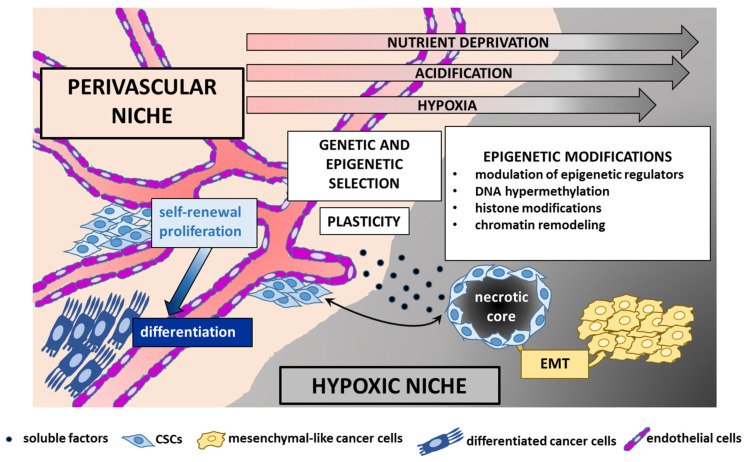
Schematic of the main factors that contribute to intra-tumoral heterogeneity in ependymoma. Genetic alterations and epigenetic modifications are selected in distinct tumor microenvironments, such as the perivascular and the hypoxic niche, that create gradients of oxygen, nutrients, and acidification. Changing contextual cues affect epigenetic regulators and remodel the chromatin landscape that mediates dynamic cellular plasticity. CSCs are able to adapt bidirectionally to both normoxic and hypoxic niches, fostering tumor growth and progression. Cross-talk among the distinct tumor and normal cell populations (such as endothelial cells) mediated by soluble factors or extracellular vesicles contributes to intra-tumoral cell-to-cell diversity. Cancer cells of the same phenotype tend to cluster together in the same topographical location. Differentiated cell concentrate in perivascular niches, whereas CSCs and mesenchymal-like cells coexist in hypoxic microenvironments, suggesting cooperative interactions and interconversion between these cell states (epithelial-to-mesenchymal transition, EMT).

Microenvironmental variability promotes commonly observed phenotypic cellular properties, such as stemness and epithelial-to-mesenchymal transition (EMT). There is an intricate interaction between CSCs and their microenvironment. CSCs are actively engaged in shaping their own supportive niche, but are in turn regulated by exogenous signals that affect their epigenome and cellular state [9,124] shaping tumor heterogeneity and evolution. Examples of the interconnections between EPN and TME are given below (Figure 4).

#### 4.3.1. The Perivascular TME

One of the key structural changes of the host tissue that cancer cells bring about to create growth promoting niches is the organization of the vascular network [3]. Variable blood flow selects for highly plastic phenotypes, whereby cells can shift from dormancy to rapid proliferation and vice versa, migrate to escape harsh contextual conditions, adapt to low oxygen concentrations, and use a wide arrays of nutrients [153,159].

In perivascular environments rich in nutrients and oxygen, cancer cells rapidly divide and foster tumor growth, whereas hypoxic contexts sustain slow-cycling stem-like cells able to drive tumor progression and recurrence due to their intrinsic plasticity and epigenetic adaptation to harsher conditions. The molecular response to hypoxia is mainly mediated by the hypoxia-inducible factor (HIF) family of TFs, especially HIF1α, that is also involved in the maintenance of stemness features and induction of angiogenesis [160]. In low oxygen concentrations, CSCs secrete a number of angiogenetic factors, such as VEGF and PDGF [161] that in turn recruit endothelial cells to shape new blood vessels, followed by CSC epigenetic adaptation to the newly formed, normoxic niche.

EPNs display remarkable spatial heterogeneity, that reflects functional adaptation to different contextual conditions. Preoperative imaging of one ST-RELA tumor showed that regions in the tumor core are associated with low blood flow as well as enrichment in hypoxia-related genes and immune-related genes characteristic of microglia, which promote an inflammatory microenvironment [136], similar to that observed in PFAs [162]. In contrast, stem-like regions are associated with high blood flow and enrichment in the histone deacetylase gene HDAC9, hinting towards chromatin modification in the cells of the perivascular areas. This is in agreement with the critical role that the perivascular niche plays for EPN CSCs, because soluble factors released from endothelial cells maintain self-renewal and proliferation of EPN SCs, while counteracting their differentiation [163].

Immunophenotyping analyses with molecular markers specific for neurodevelopmental cell types have provided the histological spatial distribution of distinct EPN subpopulations and their possible reciprocal interactions. In general, differentiated ependymal-like cells and immature stem-like cells exhibit mutually exclusive localization with a patterning in clusters of cells of the same phenotype [164,165]. Immature cells tend to concentrate in perivascular or perinecrotic zones, often colocalized with mesenchymal-like cells, suggesting a potential supportive cross-talk between both cell types. In vitro, hypoxic conditions trigger the switch of EPN cells to a mesenchymal phenotype, with upregulation of stress-related programs, including angiogenesis [164]. Hence, it is tempting to speculate that mesenchymal-mediated vasculogenesis likely occurring in vivo may sustain a perivascular niche that supports EPN neoplastic populations.

#### 4.3.2. The Hypoxic TME

Besides perivascular niches, the brain TME includes hypoxic areas [166], which result from inadequate vasculature and/or rapidly dividing cells that outstrip the local supply of oxygen and nutrients living behind many dying cells. Hypoxic areas are often acidic [167], although acidosis can also occur independently from hypoxia [168].

Studies in NSCs and embryonic neurodevelopment have supported the notion that the neural niche is relatively hypoxic, because the partial pressure of oxygen near the ependymal surface—where NSCs reside—is low [169], and hypoxic conditions in vitro maintain self-renewal and an undifferentiated state of NSCs [170]. However, perivascular [163] and hypoxic [160] areas have been reported to promote expansion and tumor-initiating properties of CSCs. These contrasting data may be reconciled by the intrinsic plasticity of CSCs, as shown in GSCs able to reversibly adapt to hypoxic and normoxic conditions [9]. Similarly, nutrient restriction enriches for GSCs able to adapt to low glucose supply by upregulating the high-affinity neuronal glucose transporter Glut3, used by cells with both a high glucose demand and a glucose-poor microenvironment, such that occurring in perinecrotic areas [171].

Hypoxia has recently been recognized as an oncogenic driver of PFA by reshaping its metabolic and epigenetic landscape [172]. Low oxygen concentration promotes optimal growth of PFA cells and induces glucose dependency and upregulation of glycolytic and hypoxia-related programs, that are prominent in PFA tumors with respect to normal brain and other EPN groups [23,151]. Hypoxia-induced epigenetic reprogramming is initiated by restrictions of metabolic intermediary products, that impact on modifications at H3K27, i.e., diminished methylation, and increased demethylation and acetylation. Mechanistically, hypoxia increases EZHIP expression, which blocks PRC2-mediated H3K27 trimethylation. Concurrently, hypoxia maintains H3K27 hyperacetylation and hypomethylation via an epigenetic mechanism that involves high levels of acetyl-CoA and metabolite-mediated activation of H3K27 histone demethylases KDM6A and KDM6B. Interestingly, a non-overlapping histological staining between the hypoxia-related marker carbonic anhydrase 9 [CA9] and H3K27me3 is observed in PFA, which underlines a link between hypoxia, metabolism, and epigenetics. Comparison with single-cell transcriptomics and metabolomics of the developing murine brain has shown that the hypoxic and glycolytic programs of PFAs mirror the signatures of gliogenic progenitors in the hindbrain that reside in low oxygen concentrations, hinting at gliogenic progenitors as the founder population of PFA [173].

Similar to findings in EPN, stress-related alterations of the homeostatic balance of chromatin via direct modulation of epigenetic regulators are observed in other cancers. In GSCs, RTK inhibition induces upregulation of histone demethylases KDM6A/B, that results in widespread redistribution of repressive H3K27me3 and chromatin remodeling and promotes drug tolerance and adaptive transition of cells to a slow-cycling state [174]. Permissive histone acetylation is under the control of heterogeneous contextual cues, such as intracellular acidification [175,176] and hypoxia [177]. In breast and lung adenocarcinoma, inhibition of the activity of oxygen-dependent TET demethylases results in DNA hypermethylation at promoters of tumor suppressor genes, associated with maladaptive oncogenic processes involved in the cell cycle, apoptosis, metastasis, and angiogenesis [178]. High TET1 levels correlating with Tet-dependent activity are observed in medulloblastoma and EPN cell lines, suggesting an involvement of TET1 in the pathogenesis of these hindbrain tumors [179].

#### 4.3.3. EMT

EMT is a highly dynamic multistep program, whereby non-motile neoplastic epithelial cells, in response to pleiotropic extrinsic signaling factors, acquire mesenchymal characteristics accompanied by loss of epithelial cell–cell junctions and acquisition of migration and invasion properties [180]. The reverse of the process—mesenchymal–epithelial transition (MET)—is associated with the reacquisition of junctional complexes and loss of migratory capacity. EMT is executed by specific TFs and epigenetic regulators [181], some of which are involved in embryogenesis, suggesting a link between cellular plasticity in embryonic development, stemness, and cancer progression [182]. The bidirectional transition between CSC and non-CSC states is functionally related to MET and EMT programs, respectively, whereby CSCs may differentiate into non-CSCs by the activation of the MET programs, and non-CSCs may undergo dedifferentiation and acquire CSC-like features and tumor-initiating potential through EMT changes [183,184].

Recent findings describe an oncogenic role for EMT in ependymomagenesis [185]. A hallmark of EMT in epithelial cells is the concurrent transcriptional repression of E-cadherin and upregulation of N-cadherin, the so-called cadherin switch [186]. Cadherin switching and upregulation of the EMT-TFs SNAI1/Snail, SNAI2/Slug, and ZEB1 is observed in PFA, PFB, and ST-RELA tumors, correlating with a shorter progression-free survival [187]. Remarkably, Wani et al. first described a mesenchymal phenotype with expression of angiogenesis, migration, and adhesion gene ontologies in a subset of infratentorial EPNs similar to transcriptomal PFA and associated with a short recurrence-free survival [188], as observed in other cancers, where EMT promotes tumor progression and metastasis and is associated with a poor outcome [182].

## 5. ITH of EPN: A Single-Cell Perspective

To date, the molecular characteristics of PBTs have mostly been informed by large-scale “omic” analyses of bulk tumors. Recently, scRNA-seq strategies have overcome these technical barriers, providing resolution of the variation of bulk samples at the individual cell scale [189]. Analysis of scRNA-seq datasets from tumor samples is performed through the following main steps: (1) distinction of neoplastic from non-neoplastic cells; (2) clustering of transcriptional profiles to identify distinct tumor subpopulations; (3) comparison of the identified signatures (a) across patients, to discover common transcriptional programs relevant to the disease, and (b) with external datasets, including bulk tumor datasets and human and mouse reference atlas datasets of developing and adult brain cell types, to clarify their biological meaning [26].

Seminal studies leveraging scRNA-seq have begun to elucidate how distinct transcriptional signatures promote malignant transformation and also delineate the developmental trajectories across diverse types of PBTs, including pHGG [65,143,173,190] and EPN [164,165,173].

### 5.1. EPN Is Composed of Multiple Discrete Neoplastic Subpopulations

**Figure 5:**
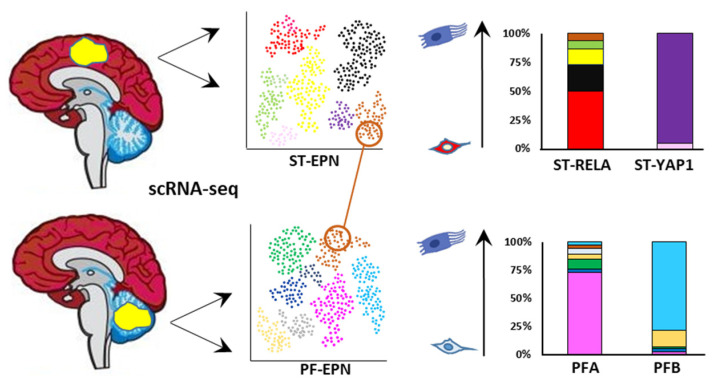
Intra-tumoral heterogeneity in intracranial ependymoma by scRNA-seq. scRNA-seq of tumor cells is colored based on distinct gene signatures that define cell subpopulations. The relative frequency of each subpopulation is different in PFA tumors that are enriched for undifferentiated cells vs. PFB tumors enriched for ependymal-like cells. ST-RELA tumors also harbor high fractions of progenitor cell subpopulations, whereas a distinct ST-YAP1 gene signature is overrepresented in ST-YAP1. Some overlap between transcriptional signatures is observed across EPN groups.

The four major childhood EPN subgroups dissected by scRNA-seq appear to be a composite mixture of multiple phenotypically discrete neoplastic subpopulations with divergent transcriptomic profiles. Although transcriptional signatures and their number differ across EPN subgroups, the common patterns of ITH that have been observed are mostly associated with cell cycle and neurodevelopmental programs. Contrasting the current classification paradigms, these studies have demonstrated that the relative proportions of the individual cell types dictate the molecular subgroup assignment, aggressiveness, and potential biomarkers of individual tumors, as reported in other brain tumors [191]. A high degree of ITH and enrichment for undifferentiated cell populations are associated with lower age and an unfavorable clinical outcome, as observed in ST-RELA and PFA, which might explain the profound difference in prognosis between these subtypes and their respective anatomical counterparts ST-YAP1 and PFB (Figure 5). Another commonality between ST-RELA and PFA is that cycling cells are specifically enriched in undifferentiated subpopulations, implying that progenitor subpopulations are more proliferative than more differentiated ones. Although, overall, cells separate according to the bulk tumor subgrouping, partially shared transcriptional programs are observed across all EPN molecular variants [164,165]. For instance, programs related to cell cycle, stress response, and ependymal differentiation are similar in ST-EPN and PF-EPN.

#### 5.1.1. PF-EPN and scRNA-seq

The more mature cell types of PF-EPN recapitulate the functional states of normal ependyma and express markers of ciliogenesis and cellular transport. Based on their function, these clusters have been named ciliated EPN cells (CECs) and transportive EPN cells (TECs) [164], and they share transcriptional commonalities with the clusters termed PF-Ependymal-like and PF-Astroependymal, respectively, in Gojo et al.’s study based on their neurodevelopmental phenotypes [165]. Both CECs and PF-Ependymal-like cells express cilia-related genes (e.g., *DNAAF1* and *RSPH1)* and TF networks, including *RFX2* [192] and *FOXJ1* [193], that play a critical role in normal ependymal development. Likewise, TEC and PF-Astroependymal display expression of the *AQP4* gene and of markers of the astrocytic differentiation lineage. AQP4 is a membrane water channel found in astrocytes and ependymal cells [194]. Increased AQP4 expression at mRNA and protein levels is observed in PF-EPN, but not ST-EPN [195,196], possibly identifying AQP4 as a compartment-specific marker of intra-cranial EPN. Other transcriptomes, referred to as mesenchymal EPN cells (MECs) [164] or PF metabolic [165], are defined by mesenchymal markers and contain stress response genes related to angiogenesis, hypoxia, and glycolysis, indicating that EMT may occur in these cells.

Undifferentiated PF-EPN cell subpopulations are distinguished by stemness transcriptional programs and markers of RGCs or early neural lineage commitment [70,100]. These clusters have been named undifferentiated ependymoma cells-1 (UEC-1) and cells-2 (UEC-2) in Gillen et al.’s study [164], and PF-Neural-Stem-Cell-like cells (PF-NSC-like), PF-Glial-Progenitor-like cells, and PF-Neuronal-Precursor-like cells [165] in Gojo et al.’s study. UEC-1 and PF-NSC-like show a broadly immature cell type and are driven by TF regulatory circuits that include JUN and FOS, which to date has never been associated with EPN tumorigenesis. Immature subpopulations in PF-EPN have also been described by Vladoiu et al. [173], who, however, failed to identify differentiated cell types, maybe due to the absence of differentiated cells in the mouse cerebellar developmental lineage comparators used.

#### 5.1.2. ST-EPN and scRNA-seq

ST-EPN is also composed of multiple neoplastic subpopulations, such as cycling cells, differentiated ST-Ependymal-like cells with a ciliogenesis-related phenotype, and less differentiated clusters that map to RGCs (ST-Radial-Glia-like) or neuronal precursors (ST-Neuronal-Precursor-like) [164,165]. Parallel to MEC and PF metabolic signatures is a cluster of cells expressing genes involved in hypoxia and glycolysis, and is named, accordingly, ST-metabolic, whereas an ST-YAP transcriptomic signature associated with more quiescent, differentiated phenotypes is highly predominant in ST-EPN harboring this fusion.

Overall, the transcriptional programs in ST-RELA subpopulations are under the control of unfused RELA activity more than ZFTA-RELA, indicating that the transcriptional programs underlying the phenotypic diversification of the ST-RELA subpopulations are independent of ZFTA-RELA activity. Of interest, whereas Neuronal-Precursor-like population in PF-EPN and ST-EPN share the NEUROG1 regulatory circuitry, which is involved in neurogenesis [197], ependymal-like populations in the two compartments show selective TF networks. FOXJ1 target genes that mediate ciliogenesis [198] are enriched in the ST-YAP cluster, consistent with a cilia-associated program hallmark of this tumor. Corroboratively, the FOXJ1 TF network also characterizes differentiated CEC and ependymal-like subpopulations in the PF compartment, specifically in PFBs, that all express a strong ciliogenesis signature [25,199].

### 5.2. The Cell of Origin and Developmental Trajectories of EPN from an scRNA-seq Perspective

All cells of the nervous system originate from a common ancestor, the NSCs lining the neural tube, the primitive developmental tissue that gives rise to the CNS [71]. In the earlier stages of normal brain development, RGCs originate from and replace NSCs at the onset of neurogenesis [200]. Unlike NSCs, the majority of RGCs are regionally and fate restricted, giving rise to a single cell type—astrocytes, oligodendrocytes, neurons, or ependymal cells—based on both extrinsic signals and cell-intrinsic factors [201]. RGCs reside in two distinct niches of the developing cortex, the ventricular zone (VZ) and the outer subventricular zone (oSVZ), and are distinguished in ventricular RGCs (vRGCs) and outer RGCs (oRGCs) by a combination of position, gene expression, cell morphology, and function [202,203].

EPNs arising in the different compartments exhibit a gene signature that recapitulates that of RGCs in the corresponding CNS region [100,188]. For instance, the human ST-EPN signature matches that of RGCs in the murine SVZ of the lateral ventricles during neurogenesis when RGCs in this region of the brain give rise to ependymal cells. Likewise, the molecular profile of spinal EPN overlaps that of RGCs in the SVZ of the spinal canal [70].

scRNA-seq and trajectory inference (TI) analyses [204,205] have shed new light on the identity of the candidate SCs and the developmental trajectories of EPN variants at different anatomical sites. Comparison of scRNA-seq datasets of EPN subpopulations to those of defined cellular lineages in the developing human and murine brain has indicated a potential cell of origin residing in the VZ for PF-EPN [164,165,173], whereas an RG-like cell residing in the SVZ is at the root of ST-RELA [70,165]. The PF-EPN and ST-EPN progenitor cells share minimal transcriptomic overlap [164], supporting that regionally and developmentally restricted populations of RGCs are the candidate cells of origin of EPNs. Across scRNA-seq studies, the putative PF-EPN cell of origin is identified in progenitor-like cells at distinct stages of neurodevelopment, and indicated as NSC-like cells [165], vRG-like cells (the predominant signature in undifferentiated UEC-1) [164], or gliogenic progenitors [173]. However, these presumptive PF founder cells share some commonalities because both NSCs and vRGCs are located in the VZ of the embryonic brain, and UEC-1 show enrichment in both gliogenic progenitor and vRGC genes.

**Figure 6:**
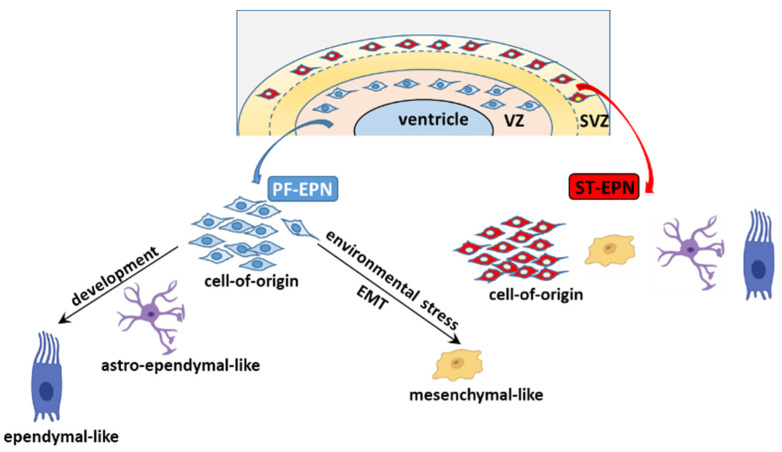
The candidate cell of origin of PF-EPNs resides in the ventricular zone (VZ) of the developing brain, whereas that of ST-EPNs is located in the subventricular zone (SVZ). In PF-EPNs, tumor subpopulations are arranged in two major distinct lineage trajectories driven by undifferentiated progenitors, that either undergo impaired differentiation towards ependymal-like cells, or transition to mesenchymal-like cells in response to cellular stresses, e.g., hypoxia. In ST-EPNs, clear developmental trajectories have not been identified yet (Reprinted with permission from Austin E. Gillen et al., (2020), Elsevier and Copyright Clearance Center, Licence Number 5200810483356, 2 December 2021).

Leveraging scRNA-seq and TI analyses, it emerges that the distinct PF-EPN subpopulations are arranged in a neural tri-lineage cancer hierarchy driven by immature progenitor cells at the apex, that undergo impaired differentiation along neuronal, astrocytic, and ependymal-like trajectories [165]. Of the three branches, the predominant one sees the NSC-like population differentiate into less aggressive progenies, the astro-ependymal cells, and successively to ependymal-like cells, presumably in response to developmental or differentiation stimuli (Figure 6). This axis potentially overlaps with the differentiating trajectory described in Gillen et al.’s study, whereby the stem cell population of UEC-1 develops into TECs (which express markers of further differentiation, such as oRGC genes, as well as gliogenic progenitor and astrocytic progenitor genes), and then CECs characterized by an ependymal-like signature. In response to unfavorable microenvironmental cues, such as oxygen and/or nutrient deprivation in hypoxic areas, undifferentiated UEC-1 develop along a stress-associated trajectory and undergo EMT to give rise to mesenchymal MECs.

The cellular make-up of ST-EPN mostly includes immature cells stalled at the earlier phases of the neurodevelopment with a minor component of more differentiated cells. Therefore, ST-EPNs appear to be characterized by the coexistence of distinct progenitor populations rather than a hierarchical developmentally organized structure.

## 6. Therapeutic Applications

Cancer cell heterogeneity has long been recognized as a major cause of treatment failure. More effective chemotherapeutic strategies should consider targeting not only driver genes, but also different cell types and cell states [206]. This is urgently needed for the aggressive forms of EPN for which effective drugs are still lacking. The distinct inter-group and intra-group transcriptomic signatures identified in EPN may help define molecular dependencies and treatment vulnerabilities.

A potentially druggable pathway in PFA EPN is EZHIP, although direct targeting of EZHIP might prove difficult to achieve, because no enzymatic activity has hitherto been identified [207]. High EZHIP expression sensitizes to PARP inhibitors by inhibiting homologous recombination-mediated DNA repair, especially in combination with radiotherapy, indicating this treatment approach as potentially beneficial in PFA [208]. A recent publication has shown that PFA tumors and patient-derived cell lines with EZHIP overexpression exhibit enhanced glycolysis and tricarboxylic acid cycle (TCA) metabolism, associated with enrichment of H3K27ac at *hexokinase-2*, *pyruvate dehydrogenase*, and *AMPKα-2* [209]. The antidiabetic AMPK activator metformin increases H3K27me3, while reducing EZHIP expression, TCA cycle metabolism, and tumor growth. As ST-EPNs also show cell subpopulations with increased glycolysis (e.g., ST-metabolic), it is conceivable to hypothesize that repurposing metformin as an antitumoral agent might have therapeutic efficacy in pediatric EPN. The opposite role of EZH2 in PFAs—whereby it is globally repressed, although its residual activity is essential for tumor development—calls for preclinical and clinical testing of EZH2 inhibitors as potential therapeutic interventions in PFA EPN. Numerous EZH2 inhibitors are currently undergoing phase 1 and phase 2 clinical testing in different tumors [210], and might have important implications for novel treatment protocols. Specifically, an advanced trial is evaluating the effectiveness of the EZH2 inhibitor tazemetostat in pediatric patients with recurrent EPN (NCT03213665).

Compounds aimed at blocking the oncogenic NF-κB pathway [211] are potential therapeutic agents against ST-EPNs harboring *ZFTA–RELA* fusion that contains the NF-κB subunit encoding gene *RELA*. The NF-κB subunit is activated via proteosomal degradation of its inhibitor IκB, thus suggesting proteasome inhibitors as candidate drugs in ST-RELA [212]. Marizomib, a pan-proteasome inhibitor with good brain-penetrating capacity [213], is currently undergoing a phase 2 clinical trial in adult patients with anaplastic EPN (NCT03727841), while in the pediatric setting it is being tested against diffuse intrinsic pontine glioma (DIPG) (NCT04341311).

scRNA-seq is expected to make significant breakthroughs in EPN and inform future therapeutic approaches. Since an increased proportion of differentiated cells is associated with a favorable clinical behavior in ST-RELA and PFA, differentiation-promoting agents might prove effective in these high-risk groups. Corroboratively, retinoids have demonstrated selective efficacy against EPN lines compared to other brain tumor-derived models in an in vitro drug screen [214].

In the context of PF-EPN, a druggable driver in the PF-NSC-like program is the Wnt pathway gene *LGR5*, a key mediator of cell proliferation and stemness features, as shown by small interfering RNA (siRNA)-mediated *LGR5* knockdown that results in reduction of self-renewal [165]. In PF-Neuronal-Precursor-like cells, targetable pathways might be the epigenetic regulators HDAC2, DNMT3A, and BRD3. Indeed, the pan-HDAC inhibitor CN133 [215] and the HDAC2 inhibitor panobinostat [165], as well as the pan-BRD inhibitors JQ1 [199] and OTX012 [216], have been reported to decrease cell viability and tumor growth in patient-derived PFA cell lines. Emerging epigenetic therapies under evaluation in clinical trials for brain tumors, including EPN, have been reviewed recently [217].

As for ST-EPN, actionable vulnerabilities in ST-Radial-Glia-like cells are FGFR3 and IGF2, whereas in the ST-Neuronal-Precursor-like subpopulation they are CCND2 and HDAC2. *FGFR3* mRNA levels are enriched in ST-RELA EPNs, and specifically in cycling and progenitor-like cell populations, mirroring *FGFR3* expression in RGCs of the embryonic and adult brain [218]. Indeed, blockade of FGFR by dominant-negative and pharmacological inhibitors impairs cell survival and stemness features in ST-RELA cells [165,218] Simultaneous inhibition of CDK4/6-CCND2 (with palbociclib) and IGF2/IGF1R (with ceritinib) pathways results in combinatorial drug efficacy, highlighting that targeting distinct subpopulations may be a successful therapeutic option [165]. CDK4/6 has also been proposed as an actionable driver in PFA, because the tumor suppressor gene *CDKN2A*, which codes for the CDK4/6 inhibitor p16, is epigenetically silenced by H3K27 trimethylation in PFA [146,207]. A phase 1 trial is addressing the safety and tolerability of the CDK4/6 inhibitor ribociclib in children and young adults with recurrent brain tumors, including EPN (NCT03434262).

A link between the overexpression of strong growth-promoting IGF2 and members of the PLAG1/PLAG1L TF family is emerging in EPN. The PLAGL1 gene is developmentally regulated and is expressed in NSCs and developing neuroepithelial cells, with low expression in the adult brain [46,219]. Although the function of PLAGL1 in tumorigenesis is controversial, acting as either a tumor suppressor or an oncogene in a context-dependent manner, PLAG1L has been shown to foster progression of GBM [207]. In ST-RELA tumors, ZETA-RELA protein binds to PLAGL family TF motifs, indicating a possible corecruitment to drive ependymoma-related transcriptional programs [106]. PLAG1 is silenced during development by PRC2-mediated H3K27 trimethylation; however, in tumors with impaired PRC2 function, such as PFA and H3K27M mutant pHGG, PLAG1 is derepressed, leading to overexpression of its downstream targets, including IGF2 [207]. Therefore, it is conceivable to hypothesize that the PLAG1/PLAG1L-IGF2 axis might be therapeutically targeted in EPN.

## 7. Concluding Remarks

Despite the enormously increased understanding of the molecular drivers and biology of EPN, the treatment standards have essentially remained static over recent years. Gross total resection is still the strongest predictor of outcome [220]. The role for chemotherapy in young children to protect them from the side effects of radiation therapy is still debated [221,222], whereas no survival advantage with the use of chemotherapy in recurrent EPN has been found [223] despite intensive investigation.

At present, 108 clinical trials are ongoing in pediatric EPN (ClinicalTrials.gov [224], accessed on 22 November 2021), however, they do not take into account EPN variants. Moving forward, the next challenge is to go beyond tumor control and to include EPN molecular classification in treatment decisions so as to adapt therapeutic strategies based on risk stratification, reducing therapy-induced morbidity in low-risk patients, while intensifying treatment for high-risk patients [225]. Development of new treatments for patients with EPN, especially in the pediatric cohort, meets several challenges, including low investment by pharmaceutical companies and low incidence of patients with rare cancers, that hamper testing of new compounds in prospective clinical trials [212]. The establishment of appropriate preclinical models, which mirror the distinct EPN subgroups and even the distinct EPN subpopulations, is critical for drug testing and identification of drug response biomarkers [226]. Although there are many preclinical studies in EPN models [74,199,227,228], few studies have hitherto compared drug sensitivities in heterogeneous subpopulations of EPN cell lines [78,79,216].

scRNA-seq has just begun to be applied to translational research in EPN. Future studies are warranted to increase the number of EPN specimens dissected at the single-cell level, to sample anatomically and temporally distinct regions in order to address tumor heterogeneity and evolution at recurrence, with the ultimate goal to discover EPN sub-type specific drivers and druggable pathways. In addition, the diverse and complex extrinsic interactions of EPN cells with the tumor microenvironment should also be prospectively evaluated.

Although single-cell genomics have shown the complex intercellular variability that governs EPN biology and challenges the response to treatment, they have also evidenced coalescing commonalities shared across subgroups of tumors and even across tumors of disparate histologies. For instance, Neuronal-Precursor-like programs are strictly correlated in PF-EPN and ST-EPN, and share transcriptional overlaps with the Neuronal-Precursor-like cell programs described in GBM [165,229]. Likewise, the mesenchymal signatures in PF-EPN and ST-EPN are similar to that reported in GBM. In addition, the astro-ependymal program in PF-EPN resembles the astrocyte-like programs in both DIPG and GBM [165,190]. In addition to scRNA-seq, integrative proteogenomics analyses have identified common biological processes between and among PBTs of seven histological types, including HGG, and EPN, which suggests that tumors of disparate histologies may share common therapeutic vulnerabilities [230].

In conclusion, not only has scRNA-seq highlighted the bewildering heterogeneity of EPN, but it may also contribute to defining subtype- and subgroup-specific molecular vulnerabilities and new options for therapeutic interventions. Moreover, the observation that some transcriptomic signatures cross histological boundaries among EPN groups and even amongst disparate pediatric tumors suggests that some treatment opportunities may be effective in a larger group of diseases than that might have been expected.

## Abbreviations

BTSCs	brain tumor stem cells
CEC	ciliated ependymoma cells
CIMP	CpG island methylator phenotype
CSCs	cancer stem cells
DIPG	diffuse intrinsic pontine glioma
ecDNA	extrachromosomal DNA
EMT	epithelial-to-mesenchymal transition
EPN	ependymoma
ESCs	embryonic stem cells
GBM	glioblastoma
GSCs	glioblastoma stem cells
ITH	intra-tumoral heterogeneity
MEC	mesenchymal ependymoma cells
MET	mesenchymal epithelial transition
NSCs	neural stem cells
NSs	neurospheres
OPCs	oligodendrocyte precursor cells
oRGCs	outer radial glia cells
oSVZ	outer subventricular zone
PBTs	pediatric brain tumors
PF	posterior fossa
PF-NSC-like	posterior fossa-Neural-Stem-Cell-like
pHGG	pediatric high-grade gliomas
RGCs	radial glia cells
RTK	receptor tyrosine kinase
RT-PCR	reverse transcriptase polymerase chain reaction
scRNA-seq	single-cell RNA sequencing
ST	supratentorial
ST_EPN	supratentorial ependymoma
TCA	tricarboxylic acid cycle
TECs	transportive ependymoma cells
TF	transcription factor
TI	trajectory inference
TME	tumor microenvironment
UEC-1	undifferentiated ependymoma cells-1
vRGCs	ventricular radial glia cells
VZ	ventricular zone
